# Emergency Ultrasound Fellowship Match Outcomes: Unfilled Positions and Implications over Eight Years

**DOI:** 10.5811/westjem.64010

**Published:** 2026-10-18

**Authors:** Zachary Boivin, Di Coneybeare, Alexis Salerno, Sandra Werner, Trent She

**Affiliations:** * Yale School of Medicine, Department of Emergency Medicine, New Haven, Connecticut; † Columbia University Medical Center, Vagelos School of Physicians and Surgeons, Department of Emergency Medicine, New York, New York; ‡ University of Maryland School of Medicine, Department of Emergency Medicine, Baltimore, Maryland; § Case Western Reserve University, MetroHealth Medical Center, Department of Emergency Medicine, Cleveland, Ohio; || Hartford Hospital, Department of Emergency Medicine, Hartford, Connecticut

## Abstract

**Introduction:**

Advanced emergency ultrasound (AEMUS) fellowships recently experienced an unexpected decrease in the number of positions filled in the match. Our study aimed to characterize this trend over an eight-year period.

**Methods:**

We performed a cross-sectional observational study of AEMUS fellowships participating in the National Resident Matching Program (NRMP) from the 2019–2026 match cycles, cross-referencing the publicly available database from the Society of Clinical Ultrasound Fellowships. The primary outcome was both the proportion of AEMUS fellowship positions filled and a program complement fill percentage. Secondary outcomes included stratification by geography and duration since program establishment.

**Results:**

We included a total of 138 of the 141 programs listed in the NRMP Match. From 2019 to 2026, there was a 28.2% increase in the number of programs and a 34.5% increase in available positions. There was a general increase in the percentage of positions filled from 2019 to 2022, with 53.7% (87/162) of positions filled to 74.4% (145/195), peaking at 81.1% (137/169) in 2021. This upward trend reversed in 2023 with a decrease to 65.1% (142/218). The percentage of positions filled has continued to decrease through 2026, with a fill rate of only 56.0% (122/218). In the eight years of NRMP data, 97 of the 138 programs (70.3%) went unfilled at least once. Programs participating in the Match for a longer period had a statistically significant higher fill percentage, with 27 programs in the 1- to 4-year group with a total fill percentage of 46.0%, 32 programs in the 5- to 7-year group with a fill percentage of 59.0%, and 79 programs in the ≥ 8-year group with a fill percentage of 72.8% (*P* < .01) using a Kruskal-Wallis test. Spearman correlation analysis found no association between geographic proximity and likelihood of a program being fully filled.

**Conclusion:**

This cross-sectional study of NRMP data showed a decrease in the percentage of positions filled for adult AEMUS fellowships starting in 2023, which continued through 2026. Programs with greater longevity exhibited higher fill percentages, showing resilience in the face of this pattern.

## INTRODUCTION

Since the first advanced emergency ultrasound fellowship (AEMUS) started in 1993, there has been widespread proliferation and acceptance of point-of-care ultrasound (POCUS) in emergency medicine (EM) and undergraduate medical education.^1^–^6^ The clinical utility of POCUS and its increasing popularity led to the implementation of POCUS training guidelines for EM postgraduate training by the Accreditation Council for Graduate Medical Education (ACGME) in 2009.^2^,^7^,^8^ In response to the growing body of AEMUS fellowship programs, the Society of Clinical Ultrasound Fellowships (SCUF) was formed to centralize the application process and provide a common virtual location where fellowships could recruit potential fellows. The AEMUS fellowships started to participate in the Match through the National Resident Matching Program (NRMP) in 2019. Subsequently, an independent fellowship accreditation body, the Emergency Ultrasound Fellowship Accreditation Council was established in 2020 to standardize the AEMUS fellowship experience. As of the 2026 AEMUS application season, there are 118 AEMUS programs actively accepting applications for new EM-trained fellows.

From 2019 to 2023, there was steady growth in the percentage of AEMUS positions filled. However, from 2023 to 2026, a high percentage of AEMUS fellowship positions went unfilled. This finding mirrors both the EM residency Match in recent years with a high rate of unfilled programs, and decreased EM job opportunities during the COVID-19 pandemic.^9^,^10^ In contrast, other EM fellowships such as toxicology, emergency medical services, and global health did not demonstrate the same pattern within this period.^11^ Limited research exists to examine the underlying etiology of this phenomenon.

The NRMP provides data on combined fill percentages (for AEMUS this includes programs accepting internal medicine, pediatrics, and family medicine residents), applicant numbers, and program-level data of positions filled compared to available positions. We sought to characterize both the proportion of AEMUS fellowship positions filled and the program complement fill percentage for EM AEMUS fellowships, along with identifying specific programs elements (geography, years participating in the NRMP Match) that correlate with fellowship position fill rate. Understanding the relationship between specific programs and their fill percentage can provide insight into the fellowship Match for both AEMUS fellowship directors and fellow applicants beyond the data provided by the NRMP.

## METHODS

### Study Design

We performed a cross-sectional observational study of AEMUS fellowships participating in the NRMP from the 2019–2026 Match cycles. This represents the total duration AEMUS fellowships used the NRMP process to match fellows. Prior cycles, which involved direct offers to candidates either on a rolling basis or on an agreed-upon offer date, were not included given changes in fellowship directors and anticipated large data gaps from incomplete records.

**Population Health Research Capsule**

What do we already know about this issue?

*The number of emergency ultrasound fellowship programs and positions has expanded in recent years, but data suggest declining fill rates in recent Match cycles.*

What was the research question?

*How have advanced emergency medicine ultrasound fellowship fill rates and program complement fill percentages changed over 8 years?*

What was the major finding of the study?

*More established programs in the Match had higher fill rates than newer programs (72.8% vs 46.0%, P < .01)*

How does this improve population health?

*Recruitment trends may help programs align fellowship positions with changing applicant demand and future emergency ultrasound workforce needs.*

We used the publicly available NRMP dataset to identify AEMUS programs participating in the match process, and cross-reference it with the publicly available database from the SCUF (https://www.eusfellowships.com/programs) to ensure accuracy. To accept new fellows, an AEMUS program must use both the SCUF and the NRMP: The SCUF manages the application process to AEMUS fellowships, and the NRMP manages the Match process. Any program that primarily accepted non-EM fellowship applicants was excluded. Program location was sorted into four regions (Northeast, Midwest, South, West) based on US census classification.^12^ The number of years programs participated in the Match was based on NRMP data from 2019 to 2026, and sorted a priori into three groups, 1–4 years, 5–7 years, and ≥ 8 years of total program existence at the time of the study. These groups were selected to compare fill percentage based on years of program existence, with ≥ 8 years representing the most established programs in the NRMP dataset, and 1–4 years being considered a newer program.

Because there is evidence that geographic factors play a factor in the NRMP residency Match, we assessed whether geographic factors would be a factor in AEMUS fellowship fill rates.^13^,^14^ We chose specifically to assess geographic proximity to other fellowships, theorizing that programs located closer together would have lower fill rates due to competition for applicants. The geographic driving distance between AEMUS fellowships was determined by using a program’s ZIP code and the open-source mapping software OpenStreetMap (https://www.openstreetmap.org). We grouped programs into academic or community-based categories based on self-identification on the program website or its SCUF website profile.

Manual review of the NRMP dataset was undertaken by study investigators (ZB, TS) who underwent a one-hour training session and were not blinded to the study question; there was a 10% random overlap in reviews of program fill percentages. No discrepancies were noted within the random overlap. This study was reviewed and deemed exempt by the institutional review board at Hartford Hospital.

### Outcomes

The primary outcome was both the proportion of AEMUS fellowship positions filled and a program complement fill percentage. The proportion of AEMUS fellowship positions filled was the proportion of available positions filled in the NRMP Match, whereas the program complement fill percentage was the proportion of individual programs that completely (all spots matched), partially (at least one spot matched), or failed (no spots matched) to fill in the NRMP Match. Secondary outcomes included fill percentage stratified by geography, total duration of program existence, and number of fellows (at least 1 fellow). Additional analyses were undertaken to compare Match data (unfilled, fully filled, partially filled) to program type (academic or community) and proximity to other AEMUS fellowship programs.

### Data Analyses

We used descriptive statistics to describe the Match outcomes, with fill percentage calculated as number of spots filled divided by available spots. A Shapiro-Wilk test of normality was applied to data for years participating in the NRMP Match and region of program location. We used a Kruskal-Wallis test with a post hoc Dunn test to compare the non-normal data of region of program location and years participating in the NRMP Match. A Spearman correlation test and multinomial logistic regression were conducted to examine the relationship between program type (academic or community), program proximity, and program complement fill percentage. We performed statistics using Microsoft Excel (Microsoft Corporation) and IBM SPSS (International Business Machines). The analyses were conducted at the level of program year; we did not model for repeated observations from the same programs.

## RESULTS

Within the NRMP dataset, 141 programs were listed; data were available for 138 programs. Three programs in the NRMP Match were not listed on the SCUF website database. Of the 138 programs, 43 were in the Northeast, 26 in the Midwest, 43 in the South, and 26 in the West. A summary of program statistics can be found in Table 1.

The overall fill percentage was 61.4% across eight years. The 2019 ultrasound fellowship Match included 92 programs with 162 available positions, and 87 positions filled (53.7%). By the 2026 Match, there were 118 programs with 218 available positions, and 122 positions filled (56.0%). This represented a 28.2% increase in the number of programs and a 34.5% increase in the number of available positions. There was a general upward trend in fill percentage from 2019 (87/162) to 2022 (145/195) (53.7% to 74.4%, respectively), peaking at 81.1% (137/169) in 2021. This trend changed in 2023 with a drop to 65.1% (142/218), which has continued to decrease in the most recent 2026 Match to 56.0% (122/218) (Figure 1).

The number of positions filled in 2019 was 87, reaching a peak of 145 in 2022; in 2026 the number was 122. This represents an increase of 40.2% of positions filled over the study duration, larger than the percentage increase in both programs and available positions.

Analysis of program complement fill percentage demonstrated variability in recruitment success. In the eight years of NRMP data, 97 of the 138 programs (70.2%) with available data have gone unfilled, meaning no fellows matched, at least once; 59 programs (42.8%) have gone unfilled two separate years. A total of 130 of the 138 programs (94.2%) have gone partially filled or failed to fill in at least one year. Only 8 programs (5.8%) have filled all positions every year. There was a large increase in the number of programs that went unfilled, from 9 in 2022 (9/98, 9.2% of programs) to 32 (32/124, 25.8% of programs) in 2023 (Figure 2). This coincided with an increase in available positions from 195 to 218, and in the number of programs from 98 to 124, while there was a stable number of applicants matching fellowship positions in 2022 and 2023 (145 versus 142, respectively).

When stratifying the NRMP aggregate Match data by region, the percentage of filled positions were as follows: 63.5% (Northeast); 60.8% (Midwest); 57.1% (South); and 66.0% (West); there was no significant difference in match rates based on geographic region in any of the pairings (*P* = .07).

We evaluated the relationship between fill percentage and years participating in the NRMP Match by dividing programs into three groups based on program longevity (ie, how long the program has existed), divided into three groups: 1–4 years; 5–7 years; and ≥ 8 years, with the longevity determined by the total number of years a program participated in the NRMP Match from 2019–2026 match cycles. This grouping was determined a priori by investigators. There were similar group sizes for the 1–4-year (27 programs) and 5–7-year groups (32 programs), with a larger ≥ 8-year group (79 programs) given the number of established programs in the NRMP dataset. The 1–4-year group had a total fill percentage of 46.0%, the 5–7-year group had a fill percentage of 59.0%, and the ≥ 8-year group had a fill percentage of 72.8%. The fill percentage finding was statistically significant in aggregate data *(P <* .01).

There were no overall trends when comparing fill percentage (combined proportion of filled positions to available positions) for programs accepting one fellow compared to multiple fellows (Figure 3), with significance noted favoring programs accepting one fellow in 2025 *(P* = .04) but no significance found in other years.

Spearman correlation analysis indicated that the type of primary affiliated hospital for a fellowship was not associated with program complement fill percentage (ρ = −0.006, *P* = .87). A multinomial logistic regression was further performed to examine the relationship between program type (academic versus community) and program complement fill percentage (fully filled, partially filled, or unfilled), and indicated that the type of primary affiliated hospital did not predict the odds of being unmatched (OR, 1.06; *P* = .64) or fully matched (OR, 1.19*; P* = .08) when compared with partially matched (Table 2).

We conducted a Spearman correlation analysis to assess whether geographic proximity and program type were associated with the likelihood of a program being fully filled. The model included the number of nearby AEMUS fellowships within 5, 10, and 25 miles, the driving distance to the closest adjacent fellowship, and program type. There was no statistical significance that other programs within a 5-mile (ρ = −0.001, *P* = .99), 10-mile (ρ = 0.02, *P* = .56), or 25-mile (ρ = 0.03, *P* = .44) radius were associated with a fully filled program complement (Table 2).

## DISCUSSION

In this cross-sectional study of an eight-year period of the AEMUS fellowship Match data, we identified four main findings that expand upon the publicly available NRMP data. There was a spike in the fill percentage during 2021 (137/169, 81.1%) and 2022 (145/195, 74.4%) followed by a downward trend from 2023 to 2026 that returned the fill percentage to pre-2021 levels (Figure 1). An overall fill percentage of 61.4% across all years is below the fill percentage of other EM fellowships participating in the NRMP Match.^11^ There has been a rise in the percentage of unfilled programs from 9.2% (9/98) of programs in 2022 to 31.4% (37/118) in 2026, but this is below the levels seen in 2019 and 2020 at 38.0% (35/92) and 32.1% (34/106), respectively. Neither program type nor proximity to other programs played a significant role in program complement fill percentage. Finally, newer programs in the 1–4-year range filled at a lower rate compared to programs of both the 5–7-year and ≥ 8-year groups over the last eight match cycles, but this result was not statistically significant when considering whether a program was unfilled, partially filled, or fully filled.

The publicly available NRMP data represents a combination of EM, internal medicine, family medicine, and pediatric ultrasound fellowships, which differs from our EM-focused study in that we excluded programs that accepted non-EM applicants. We expanded upon the NRMP data by providing fill percentages categorized by region and program length, along with specific data on the number of unfilled and partially filled programs, and assessing for relationships between program type and geography. These additional findings, while not significant, suggest that other factors likely influence both a program’s fill percentage and program complement fill percentage.

Despite the increase in AEMUS fellowships, the fill rate has had the opposite trend in recent years. There was a large increase in the number of programs that were unfilled compared to 2022 despite a stable number of matched applicants. This finding mirrors the EM residency Match in 2023, which saw a high rate of unfilled programs attributed to specialty burnout, the projected workforce surplus by 2030, and an increase in residency positions.^9^ However, the data also demonstrate a high percentage of unfilled programs in the 2019 and 2020 Matches. The highest percentage match occurred in 2021, with 81.1% of positions filled and 13.9% of programs unfilled, which may have been related to the weakened EM job market. While there has still been a general decline in fill percentage from 2022 through 2026, the number of matched fellowship positions has remained relatively stable. The 30% rise in the number of programs during the eight-year study period, mostly occurring between 2022 and 2025, outpaced fellowship applicants over the same period.

The lower fill percentage for more recently established programs compared to long-standing programs was possibly due to factors such as program reputation, existing program structure, and alumni network. However, the resilience of programs participating in the Match for a longer period may reflect a broader trend seen in other fellowship disciplines with longer histories than AEMUS. As a relatively newer subspecialty, AEMUS may be more susceptible to fluctuations in job prospects, global health crises, and shifts within the healthcare system.

There are several potential reasons for the proliferation of ultrasound fellowship programs and positions. There has been an increase in ultrasound education in both undergraduate medical education and residency as the use of POCUS increases, leading to an increased need for ultrasound fellowship-trained faculty.^2^ The ACGME recognized this growth and designated POCUS as a core competency for EM in 2012, mandating POCUS training with a minimum of 150 POCUS examinations before graduation.^15^,^16^ The AEMUS fellowships were seen as highly desirable for both residents pursuing fellowships and for chairs hiring fellowship graduates.^17^,^18^ Graduates of AEMUS fellowships reported high satisfaction with their fellowship training, with 93% of surveyed graduates reporting that they would recommend POCUS fellowships to trainees, and 94% of trainees stating that they would pursue an AEMUS fellowship if offered the opportunity.^19^ As POCUS became more integrated into EM residencies, medical schools and other fields started building POCUS curricula and certifications.^20^–^26^ Given this increased popularity of POCUS, there was a natural and expected increase in the demand and, consequently, the supply of AEMUS fellowships. The concern, however, is the continued high prevalence of unfilled programs. Although our data do not point to any causative reasoning for these findings, we suggest a few hypotheses.

First, the static growth of new AEMUS fellowships starting in 2023 was likely related to accreditation standards for these training programs. Despite standardized POCUS training in EM residencies,^27^,^28^ AEMUS fellowships largely operated without significant oversight, which led to variation in education. This lack of oversight was addressed by the creation of the Emergency Ultrasound Fellowship Accreditation Council to accredit AEMUS fellowships based on a set of core guidelines and content.^29^,^30^ The American Board of Emergency Medicine (ABEM) subsequently developed the Focused Practice Designation (FPD) for AEMUS providing certification for graduates of Emergency Ultrasound Fellowship Accreditation Council programs.^30^ The need for AEMUS fellowships to meet accreditation requirements potentially limited fellowship growth as preexisting programs had to remain compliant with the accreditation council’s guidelines and new fellowships underwent the rigorous accreditation process.

The increase in unfilled programs and falling Match rate in 2023 could relate to COVID-19, as the EM job market during the early pandemic was strained.^10^,^31^ The 2021 fellowship Match had the highest percentage of positions filled over the eight-year period, as residents may have pursued an AEMUS fellowship hoping to become more attractive candidates for positions in a strained job market. The subsequent decrease in Match rate over the coming years, approaching the pre-2021 fill percentage, could reflect a return to normal for the EM job market and AEMUS fellowships. Pursuing an additional year of training, at a lower salary compared to attending physicians, combined with substantial educational debt and repayment obligations may reduce residents’ interest in fellowship. Another consideration is that the increase in AEMUS fellowships may have resulted in a saturation of academic job opportunities for graduates.^32^ Since the adoption of POCUS as a core curriculum of EM residencies, more POCUS examinations with increasingly complex POCUS topics are taught earlier in a physician’s training.^2^,^33^

Overall, the rise in the number of AEMUS fellowship positions available, along with data showing that almost 95% of programs have gone unfilled or partially filled in the last eight years, suggests that the competition for programs to match applicants has increased. To attract fellows, a program may need to distinguish itself through various means including salary, number of shifts, educational and research opportunities, continuing medical education stipend, availability of advanced ultrasound procedures such as transesophageal echocardiography and nerve blocks, national and international teaching opportunities, or other factors. It also means that AEMUS fellowships that solely employ board-certified or board-eligible emergency physicians via ABEM may consider extending their draw to other boarded specialties such as pediatrics, family medicine or internal medicine. Internal medicine graduates of AEMUS fellowships may now take the FPD examination, and other specialties may follow, increasing the value in AEMUS fellowships offering non-EM positions. These findings may also be useful to organizations considering starting an AEMUS fellowship and helping existing fellowships determine number of positions.

## LIMITATIONS

This study used a cross-sectional design based on reviewing publicly available data. One limitation is that the list of fellowship programs generated may not have included those that closed during the eight-year period, affecting the fill percentage and number of positions matched by applicants. Additionally, there were repeated observations within our study across years, as programs appeared across multiple NRMP Matches. This was not statistically accounted for in our modeling and could have affected the results. We showed the effect of more years participating in the Match and its relationship to fill percentage in our results.

Neither were there data on how many fellowship applicants did not match, resulting in an underestimation of the number of residents pursuing an ultrasound fellowship. Some programs were listed with dashed lines on the NRMP sheet for years, indicating they did not participate in the Match, despite being fully accredited. These programs could have lost funding or decided not to participate in the Match. Finally, this dataset does not include candidates who filled an AEMUS fellowship position after the NRMP Match period. This would not be reflected in the fill percentage we calculated from the NRMP data and would have resulted in a higher fill percentage along with fewer unfilled programs.

## CONCLUSION

This cross-sectional study of publicly available NRMP data showed growth in available AEMUS fellowship positions from 2019 to 2026, with fill percentages peaking in 2021 and 2022 before declining from 2023 through 2026 to levels comparable to the pre-2021 baseline. Programs participating in the NRMP Match for more years showed resilience in the face of this pattern. Continued attention to these trends can help fellowship directors, leadership, and faculty focus on the changing needs and demands of the aspiring AEMUS fellow.

## Figures and Tables

**Figure 1:**
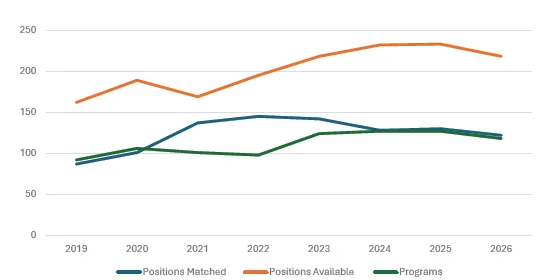
Trend of positions matched, positions available, and program number during the eight years of National Residency Matching Program data for the advanced emergency ultrasoumd fellowship Match.

**Figure 2:**
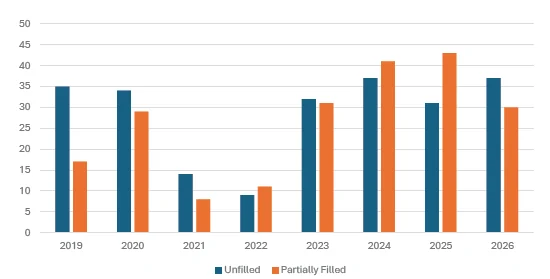
Number of advanced emergency ultrasound (AEMUS) fellowship programs unfilled or partially filled by year in a study of eight years of fill percentages in the AEMUS fellowship Match.

**Figure 3:**
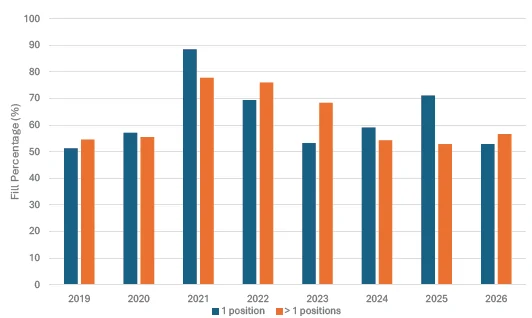
Combined fill percentage for programs with 1 position compared to > 1 position in a study of eight years of fill percentages in the advanced emergency ultrasound fellowship Match, with statistical significance in 2025.

**Table 1:** Summary of program statistics in a study of fill percentages in the advanced emergency ultrasound fellowship match.

	2019	2020	2021	2022	2023	2024	2025	2026
Positions Filled	87	101	137	145	142	128	130	122
Positions Available	162	189	169	195	218	232	233	218
Fill Percentage (%)	53.7	53.4	81.1	74.4	65.1	55.2	55.8	56.0
Program Number	92	106	101	98	124	127	127	118
Fully Filled, *n* (%)	40 (43.5)	43 (40.6)	81 (80.1)	78 (79.6)	61 (49.2)	49 (38.6)	53 (41.7)	51 (43.2)
Partially Filled, *n* (%)	17 (18.5)	29 (27.4)	8 (7.9)	11 (11.2)	31 (25.0)	41 (32.3)	43 (33.9)	30 (25.4)
Unfilled *n* (%)	35 (38.0)	34 (32.0)	14 (13.9)	9 (9.2)	32 (25.8)	37 (29.1)	31 (24.4)	37 (31.4)

**Table 2:** Summary table of statistical comparisons for geographic region, program longevity, program type, and program proximity.

Characteristic	Groups Compared	Fill Percentage / Outcome	*P* value
Geographic region	Northeast	63.5%	
	Midwest	60.8%	
	South	57.1%	
	West	66.0%	.07
Program longevity	1–4 years	46.0%	
	5–7 years	59.0%	
	≥ 8 years	72.8%	**< .01**
Number of positions	1 position vs > 1 positions	Significant difference observed only in 2025	**.04**
Program type	Academic vs Community	No association (ρ = −0.006)	.87
Proximity to other programs	Programs within 5 miles	No association (ρ = −0.001)	.99
	Programs within 10 miles	No association (ρ = 0.019)	.56
	Programs within 25 miles	No association (ρ = 0.026)	.44

*P* values are bolded if statistical significance was found.

ρ = Spearman correlation coefficient.
